# Anti-Gouty Arthritis and Antihyperuricemia Effects of Sunflower* (Helianthus annuus)* Head Extract in Gouty and Hyperuricemia Animal Models

**DOI:** 10.1155/2017/5852076

**Published:** 2017-08-27

**Authors:** Lanzhou Li, Meiyu Teng, Yange Liu, Yidi Qu, Yuanzhu Zhang, Feng Lin, Di Wang

**Affiliations:** ^1^School of Life Sciences, Jilin University, Changchun 130012, China; ^2^Jilin JiCe Testing Technology Co., LTD, Changchun 130012, China; ^3^Zhuhai College of Jilin University, Jilin University, Zhuhai 519041, China

## Abstract

This study was performed to investigate the therapeutic effects and possible mechanisms of sunflower* (Helianthus annuus)* head extract (SHE) on gout. First, the components of sunflower head powder and SHE were analyzed systematically. SHE, especially SHEB (extracted with 20% ethanol and 80% double-distilled water), strongly suppressed the swelling of the ankles in rats with acute gout induced by monosodium urate (MSU) crystals and reduced the levels of uric acid and xanthine oxidase (XO) in mice with hyperuricemia induced by oteracil potassium and yeast extract powder. Hematoxylin and eosin staining indicated that SHEB reduced inflammation cells and increased the joint space in the ankle compared with the control rats with MSU-induced gout. In the rats with acute gout, among 13 detected inflammatory cytokines, SHEB significantly enhanced the serum levels of interleukin-10 and the monocyte chemoattractant protein 1*α*. In the mice with hyperuricemia, SHEB reduced the levels of glutathione peroxidase, superoxide dismutase, malondialdehyde, and nitrogen monoxide in liver tissues. The potential therapeutic effects of SHE on gout are probably due to the production of anti-inflammatory cytokines and the suppression of XO activity via the modulation of oxidative stress status.

## 1. Introduction

Gout is a common arthritic disease associated with joint pain, fatigue, and high fever [1]. The prevalence of gout is estimated at 2%; it is especially observed among men over 40 years of age with concomitant metabolic syndrome [2]. The increasing trend of gout is likely to lead to increasingly large social costs, including direct costs related to medical treatment and indirect costs associated with absenteeism and presenteeism [3].

It is generally agreed that gout is caused by the deposition of monosodium urate (MSU) crystals within joints characterized by chronic hyperuricemia (serum uric acid [UA] levels of >6.8 mg/dL) [4]. UA is the end product of purine catabolism under the catalysis of xanthine oxidase (XO) [5] and serves as the main clinical biochemical index of gout [6]. Intracellular oxidation increases with UA production. Oxidative stress is derived from a large number of highly reactive molecules [7] and leads to imbalance in the oxidative and antioxidative systems, ultimately damaging cellular functions [8]. In contrast, UA links the symptoms of gout with inflammatory responses. MSU crystals stimulate monocytes to produce tumor necrosis factor alpha (TNF-*α*) and interleukin-1*β* (IL-1*β*) and activate endothelial cells [9].

Based on its pathogenesis, gout could be treated by reducing serum UA and dissolving urate crystals. Allopurinol (AL) is used to treat hyperuricemia but is ineffective for treating acute gout [10]. Nonsteroidal anti-inflammatory drugs (NSAIDs) exert their anti-inflammatory and analgesic effects mainly by reducing the level of cyclooxygenase [11]. Colchicine (COL), an alkaloid isolated from the lotus and seeds of* Colchicum autumnale*, reduces inflammation and the deposition of UA crystals and is mainly used to treat acute gout [12]. However, NSAIDs can cause intestinal lesions and increase the risk of kidney problems [13]. Chronic COL administration leads to neutropenia and anemia; more severe COL toxicity may result in convulsions, coma, multiple organ failure, and even death [14]. It is thus necessary to search for new alternative agents with few adverse effects for the treatment and prevention of gout.

Natural products have become a source of novel pharmaceuticals due to their potent efficacy with fewer side effects, which relies on the containing of complex bioactive compounds [15]. Quercetin isolated from* Biota orientalis* reduces UA in hyperuricemia mice caused by oxonate, which is partly due to its inhibition on XO activity in the liver [16].* Ginkgo* Folium suppresses XO activity and shows anti-inflammatory effects in the model of gout and arthritis induced by MSU crystals [17]. Sunflower* (Helianthus annuus)* head has medicinal and edible value. A sunflower head decoction has been used for infection and immunity regulation [18]. Polysaccharide obtained from sunflower head scavenges hydroxyl free radicals and inhibits the super oxygen anion in humans [19]. Encouragingly, sunflower head can successfully alleviate pain and inflammation by inhibiting the activity of cyclooxygenase-2 (COX-2), and restraining prostaglandin E2 (PGE2) synthesis and accumulation and local inflammatory cell infiltration [20]. Until now, there have been no research reports of the anti-gouty arthritis and antihyperuricemia effects of sunflower head and their possible underlying mechanisms.

In this study, we systematically analyzed the components of sunflower head ethanol extracts and examined the anti-inflammatory effects in rats with acute gout in which MSU crystals were injected and the antihyperuricemia effects in mice with hyperuricemia induced by oteracil potassium. The underlying mechanisms related to oxidative stress and inflammation were further investigated.

## 2. Materials and Methods

### 2.1. Sunflower Head Extracts (SHE) Preparation

10 g of Sunflower head powders (Collected from Baicheng, Jilin, China, in October 2015) was reflux extracted with 300 mL of double-distilled (DD) water that contained 0%, 20%, 40%, 60%, 80%, and 100% ethanol at 200°C for 1 hour, and named as SHEA, SHEB, SHEC, SHED, SHEE, and SHEF, respectively. The contents of protein, polysaccharide, reducing sugar, flavonoid, alkaloid, triterpene, and mannitol in sunflower head powders and sunflower head extracts were determined by Kjeldahl method [21], phenol-sulfuric acid method [22], 3,5-Dinitrosalicylic acid colorimetric method [23], Rutin standard colorimetry [24], Berberine standard colorimetry [25], oleanolic acid standard colorimetric [26], and mannitol standard colorimetry [27] according to previous studies.

### 2.2. Experiments on MSU Crystals-Induced Acute Gout in Rats

The animal protocol was approved by the Animal Ethics Committee of Jilin University (Reference number 2016-005). 81 male Sprague Dawley rats (8 weeks old: 180–220 g weight), supplied by Norman Bethune University of Medical Science Jilin University, Jilin, China (SCXK(JI)-2015-0003), were housed in plastic cages and maintained under standard laboratory conditions of 23°C ± 1°C, relative humidity of 55%, and 12-h light/12-h dark cycle (lights on 7:00–19:00 h) during the study. The animals were given standard rat pellets and tap water ad libitum.

#### 2.2.1. The Development of Acute Gout Rats by MSU Crystals Injection

**Figure 1:**
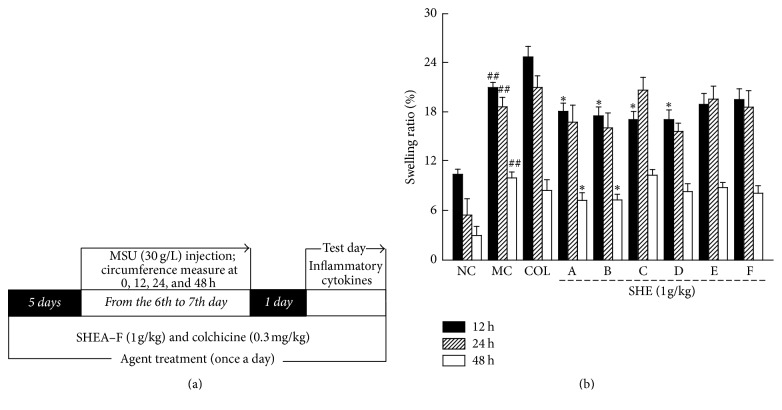
(a) The experimental protocol and drug administration in MSU crystals-injected rats. (b) The effects of SHEA–F on the swelling rate of ankle-joint in MSU crystals-injected rats. Data are expressed as mean ± SD (*n* = 9) and analyzed by using a one-way ANOVA followed by post hoc Dunn's multiple comparisons test. ^##^*P* < 0.01 versus control rats; ^*∗*^*P* < 0.05 versus model rats. NC: normal control, MC: model control, COL: colchicine, and SHE: sunflower head extract.

MSU-induced gouty arthritis rats were applied to evaluate the effects of SHE on gouty arthritis similar as previous studies, with some modifications [28]. Rats were randomly divided into nine groups (*n* = 9), orally administrated with the same volume of saline, served as control group (NC) and model group (MC), 0.3 mg/kg of colchicine (COL; positive control group) (Yunnan Phytopharmaceutical Co. Ltd, Yunnan, China), and 1 g/kg of SHEA–F (SHE-treated groups) orally administrated for 8 days. At the 6th day, all rats except for control group were injected with 3 mg of MSU (Sigma-Adrian, USA) (dissolved in normal saline) into the right ankle synovial space 1 h prior to daily gavage. At the 9th day, 1 h after the final agents administration, blood was sampled form caudal vein of rats. Serum was separated and stored at −80°C until biochemical detection (Figure 1(a)).

#### 2.2.2. Swelling Ratio Measurement

The right ankle circumference of all rats at 0, 12, 24, and 48 h after MSU injection was measured by vernier caliper. The swelling ratio (%) is calculated according to the change of the circumference following the formula: swelling ratio (%) = (*C*_*t*_ − *C*_0_)/*C*_0_, wherein *C*_*t*_ represented the circumference at different times and *C*_0_ represented the circumference at 0 hour.

#### 2.2.3. Histopathological Assessment of Ankle Joints

After sacrifice, the right ankles were collected and fixed in 4% paraformaldehyde and decalcified using 10% ethylenediaminetetraacetic acid. They were then dehydrated by processing in different grades of alcohol/xylene mixture and embedded in paraffin wax. The histological sections were later stained with hematoxylin and eosin for observation under the light microscope (200x). The histopathological changes were analyzed in terms of diminished joint space, deformation of joint synovium, and infiltration of inflammatory cells at the joints.

#### 2.2.4. Biochemical Assay

The serum levels of monocyte chemoattractant protein 1 (MCP-1, 41640), macrophage inflammatory protein 1*α* (MIP-1*α*, 41645), C-X-C motif chemokine 10 (CXCL10, 41570), interleukin-1*α* (IL-1*α*, 41734), IL-1*β* (43360), interleukin-2 (IL-2, 41733), interleukin-6 (IL-6, 41731), interleukin-8 (IL-8, 41716), IL-10 (41736), interleukin-17 (IL-17A, 43368), TNF-*α* (41721), prostaglandin E2 (PGE2, 41609), and interferon gamma (IFN-*γ*, 41739) in rats were determined by ELISA method using related ELISA Kits (Yuanye Bio-Technology Co. Ltd, Shanghai, China) according to manufacturer's instructions.

### 2.3. Experiments on OXO-Induced Hyperuricemia Mice

The animal protocol was approved by the Animal Ethics Committee of Jilin University (Reference number 2016-008). 81 male BALB/c mice (8 weeks old: 18–22 g weight), supplied by Norman Bethune University of Medical Science Jilin University, Jilin, China (SCXK(JI)-2015-0003), were housed in plastic cages and maintained under standard laboratory conditions of 23°C ± 1°C, relative humidity of 55%, and 12-h light/12-h dark cycle (lights on 7:00–19:00 h) during the study. The animals were given standard rat pellets and tap water ad libitum. All efforts were made to minimize animal suffering and to reduce the number of animals used.

#### 2.3.1. The Development of Hyperuricemia Mice

Unlike humans, UA can be metabolized into allantoin in mice. The hyperuricemia mouse model was established by using uricase inhibitor and large amounts of purine, with some modifications [29]. Mice were divided into nine groups randomly (*n* = 9) and orally administrated with the same volume of saline, served as control group (NC) and model group (MC), 20 mg/kg of allopurinol (positive control group) (Shimao Tianjie Pharmaceutical Co. Ltd, Jiangsu, China), and 1 g/kg of SHEA–F (SHE-treated groups) for 8 days. 20 g/kg of yeast extract powder was gavaged 12-h prior to AL and SHE administration except for control mice. From the 6th to 8th day, 1-h before AL and SHE administration, 300 mg/kg of OXO (Sigma-Adrian, USA), dissolved in normal saline, was intraperitoneally injected to mice except for control mice. 1 h after the last administration, blood was sampled form caudal vein of mice, and liver and kidney tissues were quickly collected (Figure 3(a)). All samples were stored at −80°C until assay.

#### 2.3.2. The Levels of UA and XO Measurement

Serum UA concentration was determined by enzymatic-colorimetric method, using a standard diagnostic kit (MAK077, Sigma-Aldrich, USA) according to manufacturer's instructions.

XO levels in serum and liver were determined using a standard diagnostic kit (MAK078, Sigma-Aldrich, USA) according to manufacturer's instructions.

#### 2.3.3. Measurement of Factors Related to Oxidative Stress

The levels of glutathione peroxidase (GSH-Px, 43390), superoxide dismutase (SOD, 43125), malondialdehyde (MDA, 43124), and nitrogen monoxide (NO, 43089) in serum, liver, and kidney of mice were determined using the ELISA Kits (Yuanye Bio-Technology Co. Ltd, Shanghai, China) according to manufacturer's instructions.

### 2.4. Statistical Analysis

All values were expressed as mean ±SD. A one-way analysis of variance (ANOVA) was used to detect statistical significance followed by post hoc Dunn's multiple comparisons test by SPSS 16.0 Software (IBM corporation, Armonk, USA). A value of *P* < 0.05 was considered to be significant.

## 3. Results

### 3.1. Composition of SHE

The protein, polysaccharide, reducing sugar, flavonoid, alkaloid, triterpene, and mannitol contents of sunflower head powder and SHEA–F were determined. With increasing ethanol concentration in the extraction solvent, the contents of protein, polysaccharide, reducing sugar, and mannitol were decreased; in contrast, the content of alkaloid was increased. Not including the sunflower head powder, the highest concentrations of flavonoid and triterpene were noted in SHEC and SHED, respectively (Table 1). The detailed methodology and related data can be found in Fig. 1S in Supplementary Material, available online at https://doi.org/10.1155/2017/5852076.

### 3.2. Effects of SHE on Acute Gout Induced in MSU Crystals in Rats

**Figure 2:**
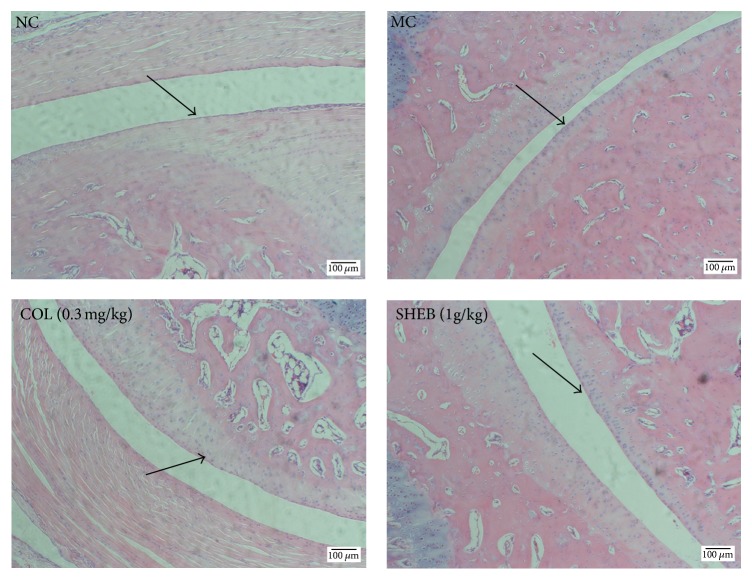
Histopathological assessment of ankle joints in rats via H&E staining observed with light microscopy (200x). Compared to normal control rats (NC), displaying normal joint microstructure, narrowed joint space, and inflammatory cells were noted in ankles of MSU crystals-injected rats (MC). COL (0.3 mg/kg) and SHEB (1 g/kg) significantly reversed these pathologic alternations in ankles of MSU crystals-injected rats. NC: normal control, MC: model control, COL: colchicine, and SHE: sunflower head extract. The arrows refer to the surface of ankle joint, the main site of deformation of joint synovium, and infiltration of inflammatory cells at the joints.

Compared with control mice, MSU injection strongly enhanced swelling of the right ankle in rats (by 21.0%, 18.7%, and 10.0% at 12, 24, and 48 h, resp.; *P* < 0.01, *F* = 24.95 to 133.78; Figure 1(b)). COL at 0.3 mg/kg failed to regulate the swelling caused by MSU (*P* > 0.05, *F* = 0.96 to 1.57; Figure 1(b)). Interestingly, SHEA–F had no suppressive effects on swelling of the ankle at 24 h (*P* > 0.05, *F* = 0.001 to 1.36; Figure 1(b)). Compared to rats with untreated acute gout, SHEA–D suppressed swelling of the ankle at 12 h (*P* < 0.05, *F* = 5.55 to 9.57; Figure 1(b)), and only SHEA and SHEB suppressed swelling at 48 h (*P* < 0.05, *F* = 4.88 to 6.30; Figure 1(b)). SHEB showed the best inhibitory effects on swelling of the ankle in MSU-injected rats and resulted in reductions of 16.2% (*P* < 0.05, *F* = 6.64; Figure 1(b)) at 12 h and 27.1% (*P* < 0.05, *F* = 6.30; Figure 1(b)) at 48 h. SHEB-treated rats were chosen for further biochemical analysis. In addition, compared to control rats, narrowed joint spaces and infiltrated inflammatory cells were found in the ankles of rats injected with MSU crystals. Encouragingly, these changes were significantly normalized by COL and SHEB treatment (Figure 2).

Inflammatory factors play an important role in the progression of gout [30]. Among the 13 inflammatory factors detected in the present experiment, MSU injection only strongly reduced the level of IL-10 (*P* < 0.001, *F* = 24.55) in the serum of rats; it was significantly recovered by administration of COL or SHEB (*P* < 0.001, *F* = 23.25 and 36.04, resp.; Table 2). Compared to control rats, no significant changes in the serum levels of MIP-1*α* were observed in rats with acute gout induced by MSU (*P* > 0.05, *F* = 0.03; Table 2). SHEB resulted in a 35.7% increase in serum level of MIP-1*α* (*P* < 0.001, *F* = 18.31; Table 2) compared to the rats with acute gout. In MSU rats, COL and SHEB showed no effects on serum levels of MCP-1, CXCL10, IL-1*α*, IL-1*β*, IL-2, IL-6, IL-8, IL-17A, TNF-*α*, PGE2, and IFN-*γ* (*P* > 0.05, *F* = 0.05 to 2.97; Table 2).

### 3.3. Effects of SHE in Hyperuricemia Mice

**Figure 3:**
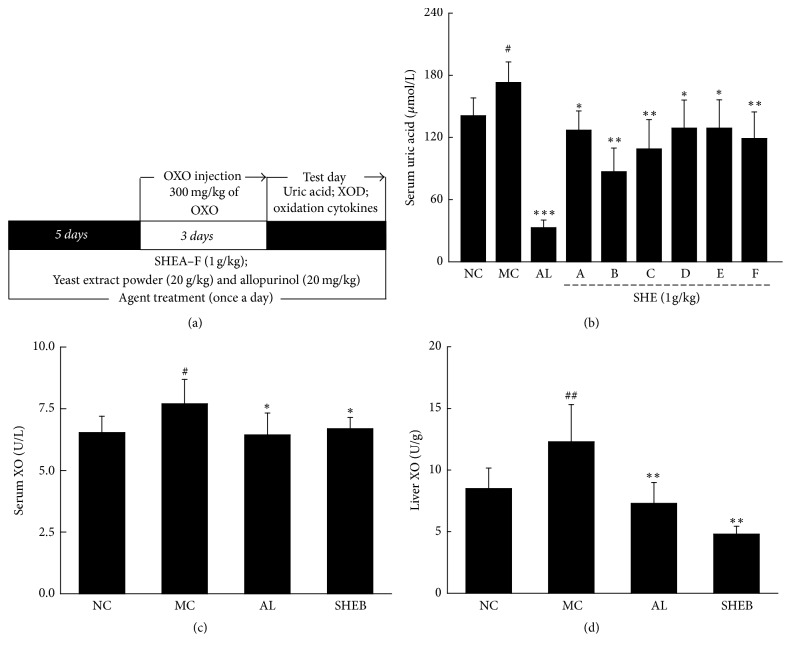
(a) The experimental protocol and drug administration in hyperuricemia mice. (b) The effects of SHEA–F on the serum levels of UA in OXO-injected hyperuricemia mice. SHEB strongly suppressed the high activities of XO in serum (c) and in liver (d). Data are expressed as mean ± SD (*n* = 9) and analyzed by using a one-way ANOVA followed by post hoc Dunn's multiple comparisons test. ^#^*P* < 0.05 and ^##^*P* < 0.01 versus normal control; ^*∗*^*P* < 0.05, ^*∗∗*^*P* < 0.01 and ^*∗∗∗*^*P* < 0.001 versus model control. NC: normal control, MC: model control, AL: allopurinol, SHE: sunflower head extract, and XO: xanthine oxidase.

Compared to control mice, strongly enhanced serum UA levels were noted in the mice with hyperuricemia (*P* < 0.05, *F* = 9.06; Figure 3(b)). Similar to AL, SHEA–F displayed significant effects on suppressing serum UA levels (*P* < 0.05, *F* = 10.47 to 58.77; Figure 3(b)), and SHEB showed the best effect among all analyzed SHE, reducing the UA serum level by 50.0% (*P* < 0.05, *F* = 58.77; Figure 3(b)). SHEB-treated mice were chosen for further biochemical analysis.

XO is the critical enzyme of purine metabolism and UA production in vivo [31]. In mice with hyperuricemia, extremely high levels of XO were noted in serum and liver tissues (*P* < 0.05, *F* = 6.97 to 13.39; Figures 3(c) and 3(d)), which were all successfully reduced by administration of AL and SHEB (*P* < 0.05, *F* = 5.63 to 60.38; Figures 3(c) and 3(d)). Compared to mice with untreated hyperuricemia, SHEB reduced XO levels in serum and liver by 13.1% (*P* < 0.05, *F* = 5.20; Figure 3(c)) and 40.2% (*P* < 0.01, *F* = 60.38; Figure 3(d)), respectively.

A great deal of reactive oxygen species are produced along with the production of UA. Antioxidative damage is considered of great importance in the therapeutic schedule for hyperuricemia in clinics [32]. In mice with hyperuricemia, no significant changes were observed in the levels of GSH-Px, SOD, MDA, and NO in serum and kidney (*P* > 0.05, *F* = 0.01 to 1.76; Table 3); in contrast, extremely high levels of these four factors were noted in liver tissue (*P* < 0.05, *F* = 5.13 to 27.88; Table 3). AL only showed suppressive effects on the levels of GSH-Px, SOD, MDA, and NO in the livers of mice with hyperuricemia (*P* < 0.05, *F* = 8.71 to 27.89; Table 3). Similarly, SHEB showed no effects on serum levels of these factors (*P* > 0.05, *F* = 0.04 to 1.61; Table 3); however, in the kidney, SHEB resulted in a 28.2% reduction in MDA concentration compared to mice with untreated hyperuricemia (*P* < 0.05, *F* = 5.04; Table 3). As shown in Table 3, in the liver, SHEB reduced GSH-Px by 65.2% (*P* < 0.001,* F *= 20.65), SOD by 59.2% (*P* < 0.001, *F* = 27.35), MDA by 66.5% (*P* < 0.001, *F* = 68.89), and NO by 59.1% (*P* < 0.001, *F* = 62.58).

## 4. Discussion

Due to unhealthy diets, the morbidity of gout is increasing year by year worldwide. In patients with gout, high levels of serum UA cause MSU deposition in joints and other tissues [33], which induces the release of proinflammatory cytokines and further promotes inflammation [34]. Based on a mouse model of hyperuricemia and a rat model of acute gout, we successfully confirmed the anti-inflammatory and antihyperuricemia effects of SHE. We found that SHEB (extracted by 20% ethanol and 80% double-distilled water) showed strong suppressive effects on synovial swelling and reduced the UA level and XO activity in serum and liver. SHEB contains various multieffective components including polysaccharide, reducing sugar, flavonoid, and alkaloid, which may target various molecules involved in inflammation signaling and oxidative stress. Systemic targeting of these molecules could completely eliminate the symptoms of gout in a much more natural way, such that few adverse effects would be expected. The safety of sunflower has been confirmed according to its traditional use [35], and our preliminary experiments on acute toxicity further confirm its safety, as indicated by nonchanging bodyweights and organ indexes in the experimental animals.

SHE successfully suppressed swelling in the ankles of rats stimulated by MSU crystals, indicating its activity against gouty arthritis. Gout occurs when the final metabolite of purine crystallizes in the form of MSU [10]. When the crystals break from tophi, they trigger an inflammatory reaction in macrophages. Our histopathologic assessment of the ankle joints in rats injected with MSU crystals further confirmed the anti-inflammatory effects of SHEB. Inflammatory and chemotactic factors are responsible for the amplification of inflammatory response and the activation of immune cells including macrophages [30]. Unfortunately, among the 13 chosen inflammatory factors, MSU crystals only influenced the serum levels of IL-10; consequently, SHEB enhanced the serum levels of IL-10 and MIP-1*α*. MIP-1*α* is a cytokine in the CC chemokine family that is involved in the acute inflammatory state via recruitment and activation of polymorphonuclear leukocytes by binding to other receptors to attract macrophages, monocytes, and neutrophils [36, 37]. IL-10, which has a chondroprotective effect, is elevated in the cartilage and synovium of patients with osteoarthritis and acts as a stimulator of chondrocyte proliferation [38]. As an anti-inflammatory cytokine, IL-10 activates macrophages to turn off, damaging the immune system during the process of gout [38]. Our data reveal that SHE shows anti-inflammatory activities in inflammation induced by MSU crystals in rats with acute gout via the regulation of cytokines, especially IL-10. However, the serum levels of TNF-*α* and IL-1*β* were not significantly changed in MSU rats in our experiment, although they were found to be enhanced in previous studies [9]. These different results may be related to the different species of rats and different times of blood collection. In our further experiments, the levels of IL-1*β* will be detected not only in serum, but also in synovial fluid.

In mice with hyperuricemia, SHEB not only suppressed the high levels of UA and XO, but also regulated the levels of factors related to oxidative stress, especially in the liver. XO catalyzes the conversion of hypoxanthine to xanthine and finally to UA [39]. Reactive oxygen species are excessively generated with UA production, leading to the overproduction of MDA and NO and promotion of SOD and GSH-Px activation by self-adjustment [40]. SOD catalyzes superoxide anions in the dismutation reaction, and GSH-Px helps to reduce lipid hydroperoxides and free hydrogen peroxide [41]. Reactive oxygen species degrade polyunsaturated lipids to form MDA, which serves as a biomarker of oxidative damage [42]. Moreover, XO can catalyze inorganic nitrite into NO by directing action and/or upregulating nitrite reductase activity [43]. Allopurinol inhibits XO activity by decreasing the concentrations of SOD, GSH-Px, MDA, and NO [43, 44]. SHEB may be a candidate for the treatment of hyperuricemia due to its inhibition of XO activation, which is partially related to its modulation of oxidative stress.

This study has limitations that will be addressed in our ongoing experiments. First, natural flavonoids and alkaloids have been reported to show potential anti-gouty arthritis properties in animal models [17, 45]. Compared to other sunflower head extracts, SHEB contained higher concentrations of flavonoid, polysaccharide, and reducing sugar. Although SHEB displayed better anti-gouty swelling and antihyperuricemia activities than other extracts, we failed to clarify which constituents of SHE were effective against gout based on our present data. As sunflower head is a natural agent, its antigout action may be related to the synergistic effects of multieffective components. Second, oxidative stress factors within inflamed joints play a major role in the pathogenesis of acute and chronic inflammation and the consequent induction of arthritis [46]. However, we studied the anti-inflammatory and antioxidative effects of SHE in different models, and the relationship between inflammation and oxidation is difficult to explain in the context of these experiments. Finally, COL, serving as the positive control drug, failed to suppress ankle edema in the acute gout rats, which may be related to the dose chosen, the duration of administration, and the rats' individual characteristics.

## 5. Conclusions

Sunflower head ethanol extracts, especially SHEB, suppress the swelling of the ankles in inflammation induced by MSU crystals in rats with acute gout and reduces serum UA levels in mice with hyperuricemia induced by oteracil potassium. Such activities are probably due to the production of anti-inflammatory cytokines (IL-10) and suppression of XO activities via modulation of oxidative stress status. Sunflower head is thus a potential agent for the treatment of hyperuricemia and gout arthritis due to its excellent pharmacological activity.

## Supplementary Material

Content determination data.

## Figures and Tables

**Table 1:** The composition and content of SHE extracted by different concentration of ethanol.

	Sunflower head powder	SHEA	SHEB	SHEC	SHED	SHEE	SHEF
Protein (%)	22.16 ± 2.32	17.79 ± 2.25	16.95 ± 2.29	15.96 ± 2.51	14.70 ± 2.46	12.18 ± 1.81	10.11 ± 1.36
Polysaccharide (%)	27.54 ± 1.64	25.28 ± 1.57	21.75 ± 1.45	19.52 ± 2.20	18.25 ± 2.12	17.13 ± 1.92	13.17 ± 0.79
Reducing sugar (%)	16.39 ± 1.42	14.66 ± 2.21	14.55 ± 1.33	14.53 ± 1.45	14.51 ± 0.96	13.86 ± 0.88	9.343 ± 1.12
Flavonoid (%) *∗* 10	14.61 ± 0.31	6.52 ± 0.08	6.68 ± 0.15	6.72 ± 0.12	6.52 ± 0.15	6.03 ± 0.14	3.43 ± 0.08
Alkaloid (%) *∗* 10	11.76 ± 0.12	0.06 ± 0.01	0.10 ± 0.01	0.22 ± 0.01	0.69 ± 0.03	1.63 ± 0.05	2.26 ± 0.07
Triterpene (%)	6.08 ± 0.22	4.39 ± 0.17	4.56 ± 0.16	5.19 ± 0.23	5.75 ± 0.21	5.70 ± 0.17	5.01 ± 0.18
Mannitol (%)	10.28 ± 0.43	8.35 ± 0.35	8.30 ± 0.55	8.00 ± 0.46	7.25 ± 0.32	6.80 ± 0.44	4.85 ± 0.32

Data are expressed as mean ± SD (*n* = 5). SH: sunflower head; SHE: sunflower head extract.

**Table 2:** The effects of COL and SHEB on the inflammation factors in MSU-induced acute gout rats.

	NC	MC	COL (0.3 mg/kg)	SHEB (1 g/kg)
IL-10 (pg/mL)	31.0 ± 4.0	22.3 ± 3.0^###^	32.9 ± 4.6^*∗∗∗*^	35.5 ± 4.9^*∗∗∗*^
MIP-1*α* (pg/mL)	453.1 ± 49.4	459.1 ± 84.6	518.3 ± 64.5	623.2 ± 69.1^*∗∗*^
MCP-1 (pg/mL)	1563.0 ± 135.5	1472.7 ± 176.4	1593.8 ± 136.4	1594.5 ± 146.5
CXCL10 (pg/mL)	32.2 ± 3.8	28.7 ± 11.4	29.9 ± 11.8	35.5 ± 3.0
IL-1*α* (pg/mL)	91.1 ± 7.5	89.8 ± 5.2	93.6 ± 6.6	88.5 ± 7.3
IL-1*β* (pg/mL)	18.6 ± 2.1	19.3 ± 2.0	18.5 ± 4.7	20.4 ± 3.7
IL-2 (pg/mL)	4044.8 ± 307.8	4107.3 ± 341.7	3826.8 ± 259.6	4284.4 ± 378.3
IL-6 (pg/mL)	708.3 ± 45.7	710.2 ± 28.9	736.1 ± 59.6	696.4 ± 43.7
IL-8 (pg/mL)	461.3 ± 28.1	479.4 ± 35.2	487.4 ± 39.3	443.9 ± 40.5
IL-17A (pg/mL)	62.6 ± 6.3	63.0 ± 7.1	64.5 ± 4.3	69.3 ± 5.9
TNF-*α* (pg/mL)	984.4 ± 71.5	953.1 ± 77.3	960.1 ± 62.7	925.8 ± 84.1
PGE2 (pg/mL)	1509.6 ± 95.9	1562.5 ± 68.8	1640.0 ± 109.1	1553.6 ± 62.5
IFN-*γ* (pg/mL)	237.1 ± 36.7	231.9 ± 20.1	244.8 ± 17.1	247.7 ± 18.8

Data are expressed as mean SD (*n* = 9) and analyzed using a one-way ANOVA followed by post hoc Dunn's multiple comparisons test. ^###^*P* < 0.001 versus control rats, ^*∗∗*^*P* < 0.01 and ^*∗∗∗*^*P* < 0.001 versus model rats. NC: normal control, MC: model control, COL: colchicine, SHEB: sunflower head extract B, IL-10: interleukin 10, MIP-1*α*: macrophage inflammatory protein 1 alpha, MCP-1: monocyte chemoattractant protein 1, CXCL10: C-X-C motif chemokine 10, IL-1*α*: interleukin 1 alpha, IL-1*β*: interleukin 1 beta, IL-2: interleukin 2, IL-6: interleukin 6, IL-8: interleukin 8, IL-17A: interleukin 17A, TNF-*α*: tumor necrosis factor alpha, PGE2: prostaglandin E2, and IFN-*γ*: interferon gamma.

**Table 3:** The effects of AL and SHEB on oxidative status in serum, kidney, and liver of hyperuricemia mice.

	NC	MC	AL (20 mg/kg)	SHEB (1 g/kg)
Serum				
GSH-Px (U/mL)	225.9 ± 26.9	218.4 ± 23.9	226.8 ± 33.6	215.0 ± 18.1
SOD (U/mL)	124.8 ± 17.2	111.9 ± 16.2	110.3 ± 15.5	110.4 ± 16.9
MDA (nmol/mL)	8.5 ± 0.8	7.9 ± 0.9	7.6 ± 1.1	7.9 ± 0.8
NO (*μ*mol/mL)	18.1 ± 2.2	19.1 ± 1.5	18.1 ± 2.5	17.8 ± 1.2
Kidney				
GSH-Px (U/g)	19541.1 ± 2253.8	20792.8 ± 4724.7	20603.9 ± 5344.4	19508.8 ± 1927.6
SOD (U/g)	11568.6 ± 1392.6	9064.7 ± 1953.8	10605.9 ± 1685.8	8242.3 ± 1082.7
MDA (nmol/g)	977.1 ± 51.7	880.5 ± 263.9	651.7 ± 129.4	633.0 ± 58.1^*∗*^
NO (*μ*mol/g)	2181.8 ± 277.5	2211.9 ± 708.9	1847.1 ± 305.4	1726.9 ± 308.8
Liver				
GSH-Px (U/g)	16049.4 ± 3777.3	24365.6 ± 8867.3^#^	12887.8 ± 4900.8^*∗∗*^	8470.9 ± 2640.0^*∗∗∗*^
SOD (U/g)	6852.8 ± 782.8	9090.2 ± 2456.2^#^	5773.1 ± 753.7^*∗∗*^	3707.1 ± 538.6^*∗∗∗*^
MDA (nmol/g)	557.9 ± 91.4	1062.4 ± 215.4^##^	503.7 ± 73.2^*∗∗*^	356.7 ± 64.6^*∗∗∗*^
NO (*μ*mol/g)	1409.9 ± 180.3	2018.2 ± 418.0^##^	1120.6 ± 234.6^*∗∗*^	826.8 ± 99.2^*∗∗∗*^

Data are expressed as mean ± SD (*n* = 9) and analyzed using a one-way ANOVA followed by post hoc Dunn's multiple comparisons test. ^#^*P* < 0.05 and ^##^*P* < 0.01 versus control mice, ^*∗*^*P* < 0.05, ^*∗∗*^*P* < 0.01, and ^*∗∗∗*^*P* < 0.001 versus model mice. NC: normal control, MC: model control, COL: colchicine, SHEB: sunflower head extract B, GSH-Px: glutathione peroxidase, SOD: superoxide dismutase, MDA: malondialdehyde, and NO: nitrogen monoxide (NO).
