# Supplementary material for: Anti-Gouty Arthritis and Antihyperuricemia Effects of Sunflower (Helianthus annuus) Head Extract in Gouty and Hyperuricemia Animal Models
Source: Biomed Res Int. 2017 Aug 27;2017:5852076. doi: 10.1155/2017/5852076 (PMC5591994; doi:10.1155/2017/5852076)
Supplement: Supplementary file 1 — Content determination data. [file 5852076.f1.doc]

**Supplementary data**

**Materials and methods**

***Content determination of sunflower head extracts and sunflower head powder***

10 g of Sunflower head powders (Collected from Baicheng, Jilin, China in October 2015) were reflux extracted with 300 mL of double distilled (D.D.) water contained 0%, 20%, 40%, 60%, 80% and 100% ethanol at 200°C for 1 hour, and named as SHEa, SHEb, SHEc, SHEd, SHEe and SHEf, respectively. The contents of protein, polysaccharide, reducing sugar, flavonoid, alkaloid, triterpene and mannitol in sunflower head powders and sunflower head extracts were determined by Kjeldahl method , phenol-sulfuric acid method , 3.5—Dinitrosalicylic acid colorimetric method , Rutin standard colorimetry , Berberine standard colorimetry , oleanolic acid standard Colorimetric and mannitol standard colorimetry according to previous studies.

**Results**

***Compositions of sunflower head extracts and sunflower head powder***

The contents of protein, polysaccharide, reducing sugar, flavonoid, alkaloid, triterpene and mannitol in sunflower head powder and SHEa-f were determined. The standard curve of Polysaccharide, Reducing Sugar, Mannitol, Triterpene, Flavonoid and Alkaloid were shown in Fig. 1s.

**References**

[1] J. Kjeldahl, “Neue Methode zur Bestimmung des Stickstoffs in organischen Körpern,” *Zeitschrift für analytische Chemie,* vol. 22, no. 1, pp. 366-382, December 01, 1883.

[2] P. S. Chow, and S. M. Landhausser, “A method for routine measurements of total sugar and starch content in woody plant tissues,” *Tree Physiology,* vol. 24, no. 10, pp. 1129-1136, Oct, 2004.

[3] J. B. Sumner, *A MORE SPECIFIC REAGENT FOR THE DETERMINATION OF SUGAR IN URINE*: McGraw-Hill, 1925.

[4] C. Zhao, X. Zhao, J. Zhang, W. Zou, Y. Zhang, L. Li, and J. Liu, “Screening of Bacillus Strains from Sun Vinegar for Efficient Production of Flavonoid and Phenol,” *Indian Journal of Microbiology,* vol. 56, no. 4, pp. 498-503, 2016.

[5] S. El-Masry, M. A. Korany, and A. H. Abou-Donia, “Colorimetric and spectrophotometric determinations of hydrastis alkaloids in pharmaceutical preparations,” *Journal of pharmaceutical sciences,* vol. 69, no. 5, pp. 597-8, 1980, 1980.

[6] Y. Chen, M.-Y. Xie, and X.-F. Gong, “Microwave-assisted extraction used for the isolation of total triterpenoid saponins from Ganoderma atrum,” *Journal of Food Engineering,* vol. 81, no. 1, pp. 162-170, 2007.

[7] L. Enbin, and C. Jianwei, “The Research of the Mannitol Contents of Changiums Myrnioides Wollf in Deferent Months,” *CHINESE ARCHIVES OF TRADITIONAL CHINESE MEDICINE,* vol. 24, no. 7, pp. 1256-7, July, 2006.


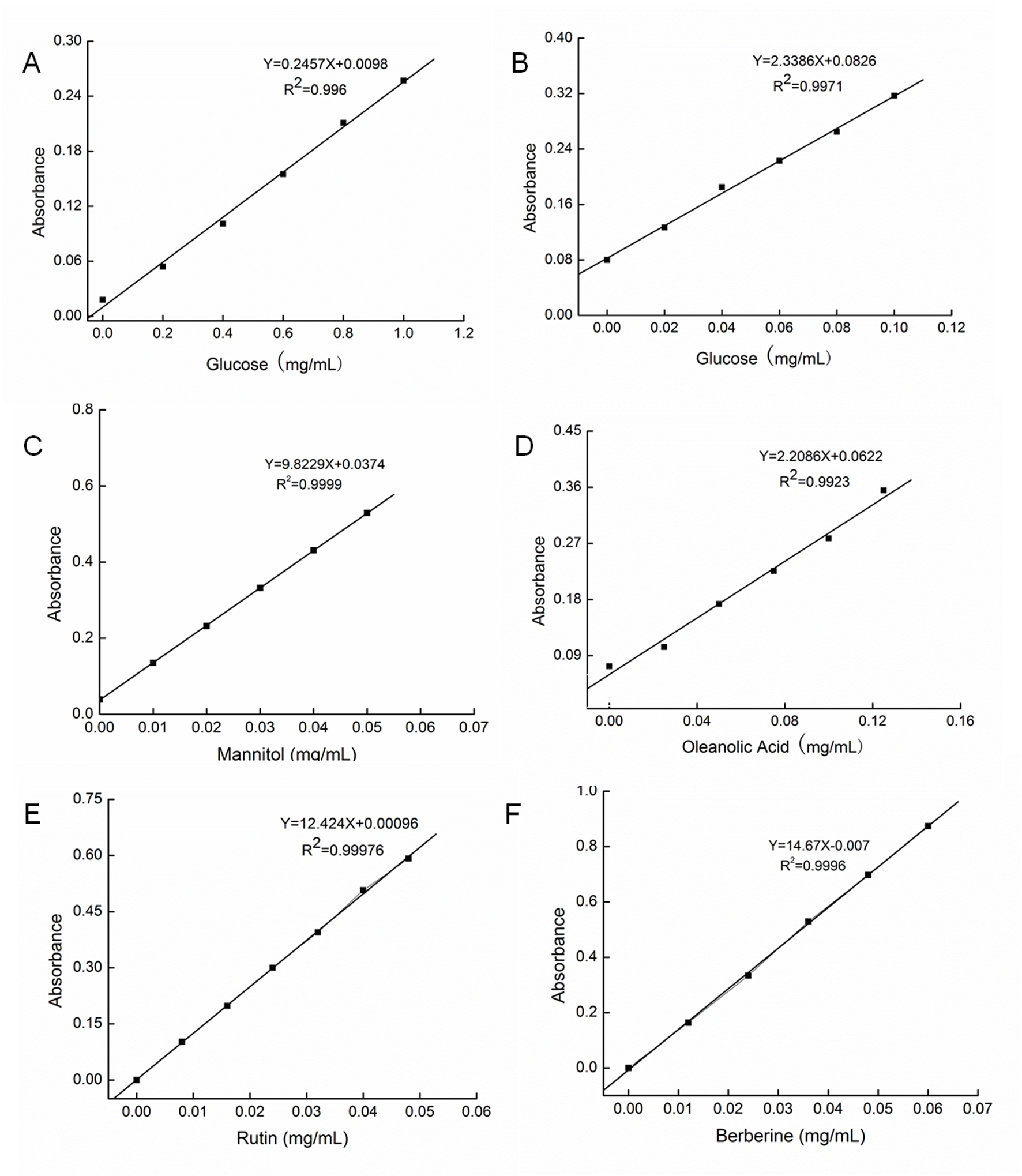


**Fig. 1s. (A)** The standard curve for polysaccharide detection. **(B)** The standard curve of reducing sugar detection. **(C)** The standard curve for mannitol detection. **(D)** The standard curve for triterpene detection. **(E)** The standard curve for flavonoid detection. **(F)** The standard curve for alkaloid detection.
